# Cases of Diseases of the Generative Organs, and of Stricture in Particular

**Published:** 1808-11

**Authors:** 

**Affiliations:** Surgeon, Edinburgh, in his Work on Gleet, Leucorrhœa, and obstinate Sores


### Cases of Diseases of the Generative Organs, and of Stricture in particular;

*Treated according to the Method proposed by* Mr. Roberton, *Surgeon, Edinburgh, in his Work on Gleet, Leucorrhœa, and obstinate Sores.*

Communicated by Mr. Roberton.

Considering the deplorable state in which the practice of Medicine as well as Surgery in many points now stands, and the impossibility, according to the present plans, either of undertstanding the real nature of disease, or the action of medicines on the body, it seemed to me that even an attempt to render these points more satisfactory and more intelligible might be of some service. It appeared to me, that a rational examination of the nature of many diseases of the generative organs in particular, has been too much neglected by medical practitioners. In consequence of this, the practice adopted in such complaints has been very often unsuccessful; and even if the original affection should have been removed, the means employed have frequently given origin to other diseases, of a more unpleasant nature, than even the one for the removal of which such treatment was applied. Of this I have witnessed frequent instances, particularly in cases of the diseases erroneously termed permanent stricture—a name now ridiculonsly applied to all diseases of these parts. The rules inculcated in many of the publications on these subjects, modern as well as ancient, are fraught with absurdity, and demonstrate at a single glance, the total ignorance of their authors respecting those laws of the animal œconomy with which every medical practitioner ought to be well acquainted, before he prescribes for any disease.

In following many of their rules, and attempting to imitate their too often confused and improbable statements, disappointment, such as happened with myself, must have been by others experienced in a variety of instances. It was the frequent repetition of such disappointments, that first convinced me of the necessity of exercising some little independence of thought in my practice in these diseases, and of only following rules, which would be supported by such reasoning as would of itself be as easily understood, as the rules which it inculcated. Thus influenced, I, some time ago, laid before the public the result of my practice in some of these diseases; and I am happy in seeing, that the rules, which I then recommended, have been found successful, and repeatedly confirmed by universal consent. Since that time, I have carried my investigations somewhat further, and have found, that the judicious management of somewhat similar rules, is equally applicable to several forms of disease, which I, in common with the public in general, had long considered irremediable.

I neither have, nor do I ever intend to publish opinions from the reports of others. I state nothing but what repeated experience and attentive observation have convinced me of; and if, from the *experience* of others, it can be proved that I have erred, I shall, with the greatest pleasure, listen to such opinions, and most assuredly confess my error. It is alone by such open, liberal, and candid conduct, that medical science can ever be benefited and improved.

Cases of gleet, of spasmodic stricture, and of debility of the generative organs, are, *in practice*, constantly confounded with each other. In the numerous volumes of those who have written on stricture in the urethra, innumerable instances of this sort are to be found; which, to particularize individually, would be a much easier than a pleasant task. I probably shall, however, at some future period, enter into an examination of the errors of the most popular books on this subject; when, I am of opinion, I shall be able to point out in a clear, and I hope satisfactory manner, many palpable mistakes and evident contradictions even in their own doctrines. *I have no hesitation in saying, that, in the course of such an inquiry into the practice inculcated in the best of these books, I shall point out simple gleet, spasmodic stricture, and debility of these parts, all treated as permanent stricture;* and, although no such disease existed previous to such treatment, shall prove that such a disease, and that too of the very worst kind, was actually induced by it, and which afterwards was, in several instances, found to baffle every attempt to remove it. Thus the wretched patient had often entailed upon him, by the wrong judgement of his surgeon, an incurable disease; and this cruel practice was either persevered in, or he was at length dismissed, with an assurance that, when neither of these existed, he either laboured under *diseased prostrate gland, or thickening in the coats of his bladder,* or something else, which could not be cured. To be reduced to such a state is particularly aggravating, when when by more rational, and much less painful treatment, the original disease might have been entirely removed.

I have also no hesitation in asserting, from very extensive opportunities of observation, that *permanent stricture* is, unless when produced by improper treatment, a disease of very rare occurrence; and when it does occur, it must be during inflammation, either from disease, or in consequence of the treatment adopted when the parts undergo the healing process, and may be induced either by inflammation of the urethra from gonorrhœa, a bruise, caustic bougies, or similar applications; never in consequence of gleet, incontinence of urine, &c. &c. which are improperly stated by authors to produce permanent strictures in these parts. Except in the false statements of those who write on strictures, we have no analogy elsewhere in the human body, of parts healing but in a certain state of inflammation; and when that is reduced below a certain degree of action, permanent stricture cannot take place. In this state, however, spasm of the parts is a very common occurrence; indeed, it is only in such a state that spasm in general can take place, at least, to such a degree as to constitute those very obstinate cases of this disease, which we so frequently meet with. Caustic, applied under these circumstances, removes the torpidity of the parts, which with more safety might have been removed by other means. Thus, inflammatory action is produced, and, by a continuance of the same treatment, aided by the strong and often violent spasm, a permanently contractile power of these parts and permanent stricture are at length produced. In many instances, however, by a continuance of these practices, inflammatory action is induced; and by thus removing the morbid action of these parts, they are, perhaps, at length, after much torment, and the utmost danger, *luckily* restored to a tolerable state of soundness.

This is the way, I believe, in which caustic is misapplied, in perhapss at least 70 or 80, probably more, cases in the hundred. In these spasmodic affections, however, which in so many instances, so much resemble permanent strictures, if we either study our own honour, or our patient's comfort, a very different plan of treatment will be adopted. I have very seldom indeed found such cases of a permanent nature, if the following plan be patiently and judiciously persevered in for a longer or shorter time, according to the severity of the complaint.

If, from a proper examination of the patient, which, though though much neglected, can and ought at all times to be made, it will be found that such a disease, for the most part, does not exist; but that spasmodic action of the parts is wholly, or for the most part, the cause of the complaint:—then the administration of large doses of antispasmodic medicines must instantly be had recourse to, and continued at least a week, when a blister ought to be applied as nearly opposite the contractile parts of the urethra as possible. The antispasmodic medicines being still continued, the blister must be kept open for several days; and, perhaps, then the warm bath may be an advisable addition to them, when a *simple* bougie, smeared with a mixture of laudanum and oil, of a size proportioned to the severity of the affection, may be introduced into the urethra, and only continued there while it does not irritate or produce pain. Thus the antispasmodic acting on the system in general, while the blister and the warm bath act on the parts particularly affected, with the occasional introduction of the bougie, when it can be done without pain, almost always cure these affections. In some indeed of a more severe nature, I have been obliged, in addition to these, to apply one blister over the sacrum and another over the pubes.

### CASE I.

On the 25th of August, 1807, I, for the first time, visited a gentleman, aged 50, who, after having exposed himself to cold and dampness, while in a state of intoxication, was, a few hours after, affected with some difficulty of voiding urine, rapidly increasing in severity.

On the 26th, he became much alarmed, and I ordered for him pills composed of camphor and opium, a scruple of camphor and two grains of opium to be taken daily. On the same evening, he passed urine in a full stream. To prevent constipation from the operation of the medicine, I likewise ordered him to use a laxative pill every night at bed time.

On the 30th, as the opium produced very obstinate constipation, I ordered him to omit it, and take the camphor by itself.

Till the 7th of September, he had no return of his complaint, except early that morning, when he was most violently seized by it. He said, that, on the previous evening, being warm and fatigued, he had drank, very rapidly, about two pints of porter, and, in about two hours after, ter, he experienced a difficulty in voiding urine. He, on the same evening, felt as if his urine flowed easily along the urethra, till it came to about half an inch from the orifice, where it stopped; and, immediately after, he felt the most excruciating pain darting along the whole course of the urethra. The spasm was so violent that, without tearing the parts, neither a bougie nor cathether could now be introduced; and as the principal obstruction seemed to be near the external orifice, I ordered, in addition to the camphor pills, a blister to be applied along the under part of the penis, from the scrotum forward, which entirely removed the obstruction, and he again passed urine freely and without pain. He observed, that a draught of cold liquor, particularly if fermented, always brought on an attack of this complaint, and that the camphor pills, with the warm bath, as regularly removed it. His whole system now became very irritable, and the slightest exposure to irregularity in living, brought back the symptoms of his complaint, at least, in a slight degree.

About midnight on the 19th, he was seized with almost complete retention of urine, which caused indescribable uneasiness, and at 7 o'clock of the following morning, when I was sent for, he could not void a drop of urine, and was nearly in a state of delirium. His bladder, however, was not distended; but, from time to time, he felt the greatest inclination to void urine, without the power of passing a drop. His pulse being very full, I took from him two pounds of blood, which exhibited strong marks of an inflammatory diathesis. I likewise ordered him a cathartic, and applied a sinapism to the perineum, and a large blister over his sacrum. In two hours after, his water came away in drops, attended with the most excruciating pain; and in twelve hours from the time I visited him, he had voided two ounces of urine. His pulse still continued full and strong, and I now took from him an additional pound and half of blood; in an hour after, he voided urine with more freedom than he had done for many hours before, though it still was attended with considerable pain.

On the 21st, the blister on the sacrum rose well, and he then voided urine with as much ease as if in perfect health. His pulse, however, was still very full. On this morning, while voiding urine, he made an attempt completely to empty his bladder, and, immediately after this, he experienced a recurrence of the spasm. To prevent this in future, ture, I desired him to make no such exertion; and ordered the cathartic to be repeated.

On the morning of the 25th, I found this patient in great agitation of mind from the dread that his complaint was about to resume its former violence. He now felt an almost constant inclination to void urine, which, uniformly, came on almost immediately after a violent passion of the mind on the preceding night, but had nearly gone off again; when, during this night, he had an involuntary emission of semen, and was immediately seized in this way. I desired him to inject some oil into the urethra, and, after taking a cathartic, to recommence the camphor pills as formerly directed. Before the evening of the same day, he voided urine freely, and in a full stream.

On the morning of the 27th, another seminal emission took place, which was immediately followed by a slight attack of spasm.

On the 28th, he followed the same plan as on the 25th, and was again well.

On the 5th of October, I made an attempt to introduce a bougie, but this could not be done without injuring the parts. The attempt was followed by partial spasmodic contractions of the urethra, and slight soreness in it for several days after.

On the 18th, after an attempt to introduce a catheter, which could not be put even within the orifice of the urethra, the spasmodic affection returned. A blister was therefore immediately applied to the perineum, and a cathartic prescribed.

On the 20th, a large sized bougie was introduced as far as two inches, but would pass no further: it occasioned no pain, and was therefore allowed to remain several hours, when it was withdrawn, and the urine flowed in a full stream.

On the 21st, a catheter of a large size was introduced as far as seven inches, and being withdrawn, a large sized bougie was then introduced and allowed to remain a few hours. It occasioned only slight pain at the orifice of the urethra, and, when withdrawn, the urine then also flowed in a full stream. A slight puriform discharge was at the same time observable, and he had a fit of shivering during the night, which greatly alarmed him.

On the 22d, he continued feverish, and could not void a drop of urine; a blister was therefore applied over the pubes, but neither catheter nor bougie could be introduced.

On the 23d, he passed urine in drops, and sometimes in a very a very small stream, which was attended with great pain along the urethra; I therefore desired the cathartic to be repeated.

On the 24th, a large bougie was introduced, which stopped at one inch and a half from the orifice of the urethra; that however easily gave way, and it passed without interruption for about six inches, where it was allowed to remain.

On the 25th, on withdrawing the bougie during the night, the urine flowed in a full stream, followed by a slight puriform discharge. It was then again introduced, and it stopped at the same place.

Early on the morning of the 26th, being affected with violent shiverings, he withdrew the bougie; they then soon went off, and he passed urine with ease, and in a full stream.

On the 27th, he himself with the greatest ease introduced the largest bougie for seven inches and a half, and permitted it to remain almost constantly till the following morning, when the shivering fit came on, and he withdrew it. The fit continued for several hours, although he applied warm fomentations, and had an injection with tinct. opii administered per anum.

On the 29th, with only a slight obstruction at two inches, the largest bougie passed easily for seven, but could not be introduced further. A small catgut bougie was, however, passed into the bladder.

On the 31st, every time he withdrew the bougie, (and it was this day introduced to within an inch of the neck of the bladder) the urine flowed in a full stream.

On the 2d of November, he felt sick, and had a slight shivering fit. I however passed a full sized bougie into his bladder, without experiencing any remarkable obstruction. It excited severe pain, particularly about the glans, and when it was withdrawn an hour afterward, the urine flowed freely, and in a full stream.

On the night of the 4th, he slept very little, and did not void urine so freely as the day before. The bougie was not again introduced till the 5th, when it was besmeared with tinct. opii and oil, and passed easily into the bladder without any other obstruction than a little at the orifice of the urethra.

From time to time this spasmodic affection returned, in a slight degree, for about six weeks, but never so severely as to prevent him from having a catheter, or a full sized bougie introduced into the bladder when found necessary.

 It

It is to be remembered that the camphor, sometimes with opium, was continued during the whole of this case.

He is now, (Sept. 1808) free from every complaint.

### CASE II.

July, 1807.—A gentleman, aged 35, of a dark complexion, and very stout, applied to me about two years before this period for the removal of a gleet, which he conceived to depend on permanent stricture of the urethra. In answer to the questions I then put, he informed me, that although the stream of urine was of a full size, it had assumed various irregular shapes, which it never had before he was affected with this complaint; but, that he passed it without any pain or uneasiness. This varied state of the stream of urine gave me reason to believe that no permanent stricture existed in his urethra, and that it was only a spasmodic affection in consequence of gonorrhœa with which he had been frequently troubled.

I did not introduce a bougie to ascertain what seemed so obvious, viz. that there was no permanent stricture present. I therefore prescribed for him the tincture of cantharides, which he used irregularly, having very little hope of being benefited by it. He soon after went to reside in London, and, anxious about the state of his complaints, he assiduously perused every book he could find that treated of strictures in the urethra; and from them having determined to use the caustic, he immediately consulted a surgeon, who judiciously advised him to begin with the daily use of the simple bougie, which was easily introduced into the urethra, and after a few attempts, one of the largest size was passed into the bladder. About two weeks after the daily introduction of the largest bougie into the bladder, the discharge from the urethra increased in quantity, and became somewhat puriform; and, upon pressure being applied, he could feel constrictions in different parts of the urethra, which he never felt before the bougie was introduced. He was now assured by his medical attendant, that the caustic bougie alone could be of service to him, and that from its use he might expect a complete cure. It was accordingly had recourse to. The first application gave him considerable pain, but it completely removed one obstruction, situated about an inch from the external orifice of the urethra. Considerable inflammation of the glans penis, with blueness and swelling of the prepuce followed in a few hours, and the discharge, during the following day, was thick and yellow, and in very great quantity. An eruption then broke out all over over his body, and watery blotches and great swelling of his face and head troubled him for several hours. This, with the blueness and swelling, abated in about two days, and the caustic was again applied; but what puzzled the gentleman was the formation of a new stricture, nearly on the same spot where the one existed, previous to the first application of the caustic. This was also destroyed, and the same effects followed as on the former occasion. By the application of the caustic bougie four times a week, for seven months, every obstruction, from the external orifice of the urethra to the bladder, was destroyed; and, all that time, a very great degree of inflammation was preserved in the parts, and consequently a puriform discharge. As new strictures were forming almost as quickly as they could be destroyed by caustic, he imbibed a notion that *he was constitutionally subject to permanent stricture.* At the expiration of seven months the whole course of the urethra was so much contracted, and the penis incurvated to such a degree, that he could not, without the greatest difficulty, introduce the very smallest caustic bougie. This was a source of much unhappiness to him, as he now found, that the introduction of this instrument was absolutely necessary, in order that a passage might be formed for the evacuation of his urine; and, from the great quantity of puriform matter which was discharged immediately after each application of the caustic, he conceived that a collection of pus had for a considerable length of time been daily forming near the neck of the bladder, for the necessary evacuation of which the caustic alone was useful. This ridiculous practice was at length luckily discontinued, and the use of the smallest simple bougie resumed, and in two, or nearly three months more, he observed that the discharge from the urethra had disappeared. Thus was he unintentionally cured of his gleet. As the spasmodic affection of the urethra, however, still continued, I desired him to use, for a short time, camphor and opium in pretty large doses, and afterward to keep the parts gently distended by the simple bougie. He then, for several weeks, adopted this practice, and experienced the greatest benefit from it.

He now says he never shall again use the caustic, as he is convinced it was owing to it that he had suffered so much; but the abettors of this practice assure him that, however much he may have suffered, he must have suffered much had he never used it, and that in expressing his opinions of the caustic bougies, he will only be ridiculed by the world, and can effect no other purpose.

 CASE

### CASE III.

A gentleman, aged 42, and very stout, was, four years ago, suddenly affected with violent retching, particularly when he coughed, to which he was very subject, or when he suddenly stooped forward. He, at that time, took medicines for the removal of these complaints, and was in a few weeks almost completely cured; but when he indulged in his *bottle*, the retching uniformly recurred, and continued to distress him for several weeks.

I was sent for in December, 1806, to prescribe for a severe, hard, dry cough, with which he had been very much troubled for some time. He had no pain in the chest, and his bowels being costive, I prescribed a cathartic, and ordered him squill pills. I likewise desired him to inhale, from a coffee or tea-pot, the steams of vinegar and warm water every night; but when he attempted to do this, the retching became very troublesome, and he was obliged to desist. Within these few days, he had felt slight pain about an inch from the orifice of the urethra; but as this affection was attended with very little inconvenience, I did not order any medicine for its removal. He had, for some time past, frequently been obliged, from torpidity in his bowels, to take cathartics; and he always observed, that the sensation in the urethra became more painful during the operation of such medicines. After he had continued the use of the squill pills for a week, without any diminution of the severity of his cough, it was deemed necessary to apply a pretty large blister to his breast, which was kept open with ung. epispast.; but even this, along with the squill pills, &c. did not seem to relieve his cough and retching in the smallest degree for more than a fortnight, when both these affections became less violent.

He was, on the evening of the 12th of January, 1807, affected with considerable difficulty in voiding urine, for which, although he applied cloths wet with warm water for several hours to the pubes and perineum, it still continued to increase in severity. At length, however, he became much easier, and slept quietly till toward morning. On examining the urine he had passed during the night, I found it completely coagulated like jelly, and he said that it had the same appearance on the preceding night, previous to his going to bed, which alarmed him greatly. He never had had any venereal complaint.

I prescribed for him pills principally composed of camphor with a small proportion of opium, so that about a scruple scruple of the camphor might be taken daily, and ordered a blister to be applied to the perineum.

On the 14th of January, the blister had not remained many hours, when he was much relieved, and voided urine freely and oftener than once during the night, which had not, as formerly, the coagulated appearance. The pain in the urethra had abated, and his other complaints were getting better.

On the 20th, when the gelatinous appearance is not present, his urine deposits a great quantity of brown sediment; but when the urine is gelatinous, it is free from sediment: and when the retching or cough is severe, the pain and difficulty in voiding urine cease, and uniformly becomes more severe when the cough, &c. cease.

On the 27th, he had a slight return of the spasmodic affection; but it went off in a few hours, without his having occasion to use any remedy.

On the 5th of February, in consequence of his complaints in the urethra having returned with considerable violence, he had again been obliged to apply the blister to the perineum, which had again relieved him; but immediately after, the cough and retching, as usual, commenced. Another blister was applied to his thorax, and I desired that both of them might be kept open with epispastic ointment. In ten days after this, the blister in the perineum healed, but that on the breast was kept open.

In May, none of his complaints had troubled him since the last report, and I desired him to allow the blister on his breast to heal. In June, 1808, he had had no return of his complaints, and enjoyed a state of excellent health.

### CASE IV.

A gentleman, aged 40, stout, and of a dark complexion, four years ago contracted a gonorrhœa, which was removed by injections, containing acet. plumbi in solution. Soon after the removal of the discharge, he felt great debility of the parts of generation, and very seldom had any inclination to the fair sex. It was not till after two years and a half that he began to recover his wonted vigor, when he again had the misfortune to contract a gonorrhœa: this too was cured by injections, containing acet. plumbi. Immediately after the removal of the discharge, all the symptoms of his former weakness affected him, and he repeatedly observed great quantities of scaly membranous

 substance substance floating in his urine, unaccompanied, however, by any gleety discharge.

On the 20th of April, 1807, he informed me of the above circumstances, and, in addition to his other complaints, mentioned, that for two months past he had frequently, while in bed, been affected with involuntary emissions of semen. After these occurrences, the stream of his urine was undiminished in size, and I now prescribed for him tinct. cantharid. ℥ß. Aq. font. ℥vi. M. A table spoonful to be taken four times a day.

On the 22d, he observed a drop of blood at the external orifice of the urethra, but experienced no pain or uneasiness from taking the medicine.

On the 24th, the membranous substances formerly mentioned, were not so plentiful as at first. I have therefore desired him to continue the cantharides.

For two months he persevered in the use of that medicine, in sufficient doses to produce slight difficulty in voiding urine. During this time, the seminal emissions gradually became less frequent, and the scaly substances, which he voided with his urine, changed from white to a yellowish colour, but did not diminish in quantity.

As I was obliged to be in London about the end of June, I left instructions with this patient, on whose accuracy I could depend, to continue the tincture in doses similar to what he had been accustomed to, and to use the cold bath thrice a week.

On my return, on the 5th of August, he informed me, that he had continued the cantharides regularly till within the last ten days; and as he had even previously to that period, experienced the most beneficial changes in his health, I declined giving him more of it. The scaly substances had now completely disappeared, and, for the last six weeks, he had only two seminal emissions. I however desired him to continue the sea-bathing while the weather was favourable.

In September, 1808, he had experienced no return of his complaints, and his general health was much better than it had been from the commencement.

### CASE V.

January 12, 1807.—A gentleman, aged 28, complained of very great general debility, with acute pain in his loins, occasionally darting down his thighs.

For fourteen years he had been subject to wet dreams, sometimes ometimes returning almost every night. He never had, however, any gleety discharge, or any venereal complaint, never having had a connection with any female. His attempts of this kind were attended with such *immediate embarrassments* as must be expected, producing the usual despondency of mind.

These emissions usually occurred during the night, while he was in bed, and were followed by a disagreeable burning heat all over his body, with great anxiety and heaviness, but a complete inability to sleep during the remainder of the night. He, however, passed his urine in a full stream.

I ordered him to substitute for the soft bed, which he had been accustomed to, a hard mattrass, to use few bed clothes, and to sleep in a well ventilated bed chamber. I likewise prescribed nourishing diet, with two or three glasses of wine after dinner, and tinct. cantharid. aq. font. of each ℥ij. M. Two tea-spoonsfull to be taken thrice a day in a glass of water.

On the 13th, he was affected with considerable pain and difficulty in voiding urine, I therefore ordered the cantharides to be taken in smaller doses.

On the 16th, he had taken the cantharides in sufficient doses to keep up a slight degree of uneasiness in the urethra, but had nevertheless an emission last night while in bed. I ordered the canth. still to be continued.

On the 20th, he had another emission. These, however, were now less frequent than before the use of the cantharides.

On the 21st, he had another emission, after which, however, the burning heat, &c. over his body, did not trouble him. The cantharides were therefore still continued.

On the 6th of February, he continued to take the canth. in sufficient doses to keep the parts uneasy, and till the morning of this day, had no emission since the 22d ult. and even it was not attended by the disagreeable symptoms above described. The cantharides were therefore still continued.

On the 7th, he had another emission. Since, however, he began to use the cantharides, he had observed that it was not till two or three days after an emission, that the medicine again produced its usual effects on the urinary organs. The emissions, however, were now of rare occurrence, and he felt stronger and in better health than he had done for several years past. The pain in his back had greatly abated. I still ordered the cantharides to be continued.

On the first of April, emissions, since last report, had occurred about once a week, and he thought they were now most frequent when he happened to take an over dose of the cantharides. I however desired him to use the cold bath twice a week, and to continue the cantharides.

On the 26th, the emissions still continued once a week, but were unattended by the disagreeable sensations which formerly accompanied them. Although his general health was very considerably improved, he began now, from the length of time which had elapsed since he expected relief from the cantharides, to be anxious about his complaints, and almost completely to despair of ever being cured of them. I ordered the canth. still to be continued.

On the 8th of May, the emissions had, for ten days past, been more frequent than usual. I began to suspect that he occasionally recurred to the original cause of his disease, and hinted to him, that with such habits he could never expect to be completely cured. He was rather offended at my suspicions, and positively assured me that I did not do him justice. I thought it right, however, to impress his mind very strongly on the occasion.

I did not see my patient till the first of June, when he again assured me, that my suspicions were erroneous; that he was now much better, having had no return for a fortnight past. This agreeable change however I attributed to my late remonstrance, and ordered the cantharides, &c. to be still continued.

This patient now went to sea bathing quarters, and I did not see him again for nearly three months, when he told me, that his complaints, during that period, had become worse, but he confessed that he had used the cantharides &c. rather irregularly for some time past. I then represented to him the danger attending the long continuance of such complaints, which alarmed him much, and he promised to be very attentive in future; and, by the closest attention to the rules laid down to him, he informed me, about the beginning of November, that his former complaint had returned only about once a month. By the end of the year he had completely recovered, and had even become remarkably stout.

### CASE VI.

January 4, 1808.—A gentleman, aged 22, stout made, but considerably emaciated, was about two years ago, (probably from from the same cause as in the last case) suddenly affected with a similar inconvenience, sometimes thrice in the course of one night, attended with an uncommon desire to venery. Since the commencement of these emissions, he had been affected with gonorrhœa, which was soon cured by the use of injections. His original complaint, however, did not seem in the least affected by the gonorrhœa, as it continued exactly in the same degree after that disease had left him.

Palpitation of the heart, and almost constant ringing in the ears, had of late troubled him very much. He suffered no pain; his mind however was in the greatest state of despondence, and his existence at length actually became a burden to him. He was at last distressed by the most dreadful dreams, which rendered him melancholy for several days after. They were principally respecting the death of some of his nearest relations; and although he was not at all superstitious, he could not prevent his mind being thus affected by them. What astonished him very much was, that he often dreamed, that he himself was just about to expire in the arms of his relations; and once or twice he actually thought that he had quitted this life. He gradually became very stupid, and unable to apply his mind to any employment, but was quite sensible of being in that state, and for some time past his feelings had become morbidly acute, which, if possible, augmented his affliction.

To these symptoms were added, about a year ago, general weakness with most distressing pains in his back; and, for six months past, he had experienced sudden giddiness and a sort of faintness, during which objects of various colours seemed to float in the air before him; and this was immediately succeeded by perspiration over all his body.

He was, about the time I saw him, frequently affected with cold sweats over his body, coldness in his feet and hands, with a great degree of coldness in his generative organs. For the last three months he had, almost every night, been troubled with very painful erections, but without his former desire for venery, which often kept him awake the whole night. He was now affected with slight tickling cough, with pains in his breast; but his expectoration was not very copious, though of a blackish colour. Every attempt to copulate had, for several months, been instantly attended with an emission, and he had uniformly observed, that if at any time the emissions did not occur for for a few days, that next time they appeared the semen was greatly increased in quantity to what it was on other occasions, and continued to be so for two or three days.

A celebrated surgeon to whom he applied, recommended strong doses of physic and daily copulation, which he assured this patient would lessen the emissions! I prescribed tinct. cantharid. ℥j. Aq. font. ℥vij. M. A table spoonful to be taken thrice a day, and the doses to be gradually increased.

On the 5th, he experienced great pain in voiding urine, which continued to increase in severity during the day. He therefore left off the medicine, and before night this symptom had abated.

On the 6th, I desired him to take the cantharides as recommended on the 4th.

On the 9th, he had no return of the pain, although he was now taking the cantharides in increased doses. Since he began the use of this medicine, he slept much better than formerly, and had almost constantly felt a strange sort of prickling sensation all over his body. I have ordered the cantharides to be continued.

On the 14th, he had suffered very little pain from the cantharides since last report, and ever since he had began the use of that medicine, he had no emissions. He, however, almost every night, felt as if the emission was just coming on, though it did not. I therefore ordered the cantharides still to be continued, with half a pint of wine per diem, and the liberal use of porter.

On the 16th, he complained that general debility had again recurred, but he had no return of the emissions. I therefore ordered the cantharides, &c. to be continued still.

On the 18th, he accounted for the above symptoms from his having exposed himself to dampness for a considerable number of hours. The cantharides, &c. were still continued.

On the 23d, he had an emision while in bed, but that had not in the least distressed or even roused him from his sleep, as he did not know of it till the morning. His general health was now much improved. The cantharides were still continued.

On the 1st of February, he had no return of the emission, and, from the changes that had taken place in his health, he was in very high spirits. He was now stout and able to walk a number of miles without being fatigued which which formerly he could not do. The cantharides were still continued.

On the 6th, he had, for two days, suffered very considerable pain from the cantharides, and during the previous night, while in bed, he had two emissions, which depressed his spirits considerably; and on the night of the 7th, he had another.

On the 8th, when he informed me of the above occurrences; he likewise mentioned, that the giddiness and dimness of sight had returned; the pain from the cantharides had however abated, and I therefore desired him to increase the doses.

On the 15th, for several hours the pain from the cantharides had been very severe, and during it he had an emission. He uniformly remarked, that he had, in rapid succession, one or more emissions when the cantharides affected him severely. These emissions, however, were not now accompanied by those troublesome sensations which formerly attended them; and his appearance was much improved having lately become much stouter than usual. Nor was he so apprehensive as formerly, and the general gloomy state of his mind was entirely removed. The cantharides were still continued.

On the 11th of March, he had since last report only one emission, which did not distress him in the slightest degree. He had now become very stout, and was, in every respect, in a state of good health. I however, desired him to continue the cantharides in moderate doses, and to use the cold sea bathing during the summer season.

In September he continued entirely free from his complaints.

### CASE VII.

20th of December, 1806, a gentleman, aged 20, apparently stout, was about 6 years ago, while at school, initiated in baneful practices. It was not till after three years continuance in these habits, that he felt the smallest inconvenience arising from them. Then however he was suddenly affected with frequent involuntary emissions while in bed; but as they proved only troublesome for the moment, he was not in the least degree intimidated by them, and for a year after he continued his habits more frequently than ever. About two years ago these involuntary emissions became more frequent, troubling him him four, and often six times every week. These were now followed by cold shiverings, which lasted for an hour or two each time, with complete inability to sleep during the remainder of the night. He was now also affected with frequent cold perspiration all over his body, with coldness of his extremities, with shrinking of his generative organs, and great pain of his stomach; sometimes with a voracious appetite for food, but more frequently a disgust to all victuals for several days; and, on such occasions, if he swallowed any thing but liquid, the sensations he felt in his stomach were not actually painful, but indescribably irksome. From the difficulty too which he felt in being obliged to perform the act of respiration, almost entirely by his voluntary powers, he believed that he should die suddenly while in bed. About that time, he felt an irregular swelling in one of his testicles, and he began for the first time to suspect the real cause of his complaint. To prevent his relations from becoming acquainted with it, he exerted his ingenuity to prevent them from applying for medical assistance. He at first thought that by abstaining from his former practices, which he now resolved to do, he should recover without being obliged to have recourse to any other expedient: but this would not do; for now, in addition to his other distresses, his bowels became obstinately costive, and he could procure no evacuation without the assistance of cathartic medicines. These, with the cold bath, and the internal use of bark and wine, and occasionally of mineral waters, were the only medicines he used till he applied to me in December, 1806.

He assured me that he had nothing to accuse himself of for about two years. His eyes however seemed dull, and he complained of partial blindness, particularly for an hour or two after he had an emission, which happened from four to six times every week. He had no gleety discharge, nor had he ever in any form been affected with a venereal complaint. He complained of an almost constant dull pain in his back, and in his stomach; and an uneasy sensation in the left testicle, which he informed me had swelled considerably for about two years. On examination I found, that this was a very considerable enlargement of the convolutions of the epididymis, and also of the spermatic chord; but as the pain from it was inconsiderable, it gave him no alarm; his bowels were still in a state of great torpidity, so that without the exhibition tion of a carthartic he was uuable to procure any evacuation; and if he omitted the liberal use of bark and wine, his food, for several hours after taking it, produced the most uneasy sensations in his stomach, and, on such occasions, he had most excruciating pains in his head.

On the 20th of December, I prescribed for him tinct. cantharid. ℥ß. aq. font, ℥vii. M. A table spoonful to be taken four times a day.

On the 23d, I repeated the mixture, and with it the following powder, as, after every dose of the cantharides, pain and uneasiness about his stomach became very troublesome. ℞. Carb. ferri, ℈j. zinzib. alb. ℈j. cort. per. a. ℥j. M. A tea spoonful to be taken thrice a day in a little water or wine.

Before a week had elapsed, he felt slight difficulty in voiding urine, but the pain in his stomach was not nearly so troublesome as before the exhibition of the powder. He remarked that all tonics, particularly of the mineral kind, had uniformly agreed with his stomach, and he thought he had derived partial relief from the internal use of such mineral waters as are to be found in the neighbourhood of this city. Emissions however still continued as formerly. On account of the pains in his stomach, I desired him to take chamomile tea instead of common tea for breakfast, and animal jellies instead of animal food for dinner; with occasionally a laxative pill, composed of equal parts of the extract of hyociamus and aloes. In this way he continued till the end of January, before he was sensible of any change in his complaints; and even then, there was only an abatement of those very distressing sensations which invariably had followed the emissions, but no alteration in the frequency of their returns.

On the 1st of February, the pains in his stomach were, immediately after the use of the tincture, very troublesome; I therefore desired him to omit it entirely, and, in addition to the other articles of diet, to use nearly a pint of port wine per diem, and instead of eating at the stated periods, to take a small quantity of animal jelly, or nourishing soup, every two hours; but never to take either of them in such quantity at once, as to satiate his appetite for food.

On the 25th, having suffered no pain in his stomach for about sixteen or seventeen days, he recommenced the use of the tincture in small doses, and his other articles of food, &c. were continued as formerly, with the addition of of the cold bath every morning. By the 12th of April, gradually augmenting the doses, he could take about three drachms per diem of the tincture, which did not occasion any pain in his stomach, but kept up a constant degree of irritation in the urinary organs. The emissions did not now occur oftener than once, sometimes twice a week, and no disagreeable sensation was felt after them when they did occur. His bowels became much more regular than formerly, as he only required one of the laxative pills every two or three days, instead of one every day.

On the 15th of May, he again felt severe pains in his stomach, immediately after taking a dose of the tincture; in consequence of which I desired him to diminish the doses, but the pain being still produced, he was ordered to discontinue the use of the tincture entirely. I remonstrated with him upon the impropriety and even danger of indulging in former practices, but he assured me that he did not, as his anxiety to be relieved of his complaints was very great. I attempted twice or thrice to recommence the use of the tincture, but even the smallest doses produced pain in the stomach; and he now observed that almost immediately after taking a dose he had an emission.

On the 1st of June, I therefore omitted the tincture, and gave him a solution of phosphorus in æther,* with directions to take two drops in a glass of water thrice a day. He once or twice added one drop to the dose more than he was desired to take; but from the disagreeable sensations which he felt in his head, immediately after taking it, he did not in future feel much inclined to deviate from the rules laid down for him; but with the greatest care and attention, continued to increase the doses till the 20th, when he told me that the emissions were now not oftener than once a week. I desired him to continue the solution, gradually increasing the dose.

In October 1807, this gentleman enjoyed much better health than he had done for about three years past; his appetite for food was more regular, and the state of his bowels more natural: his sleep was undisturbed by frightful dreams, and the emissions occurred only once a week, and were unattended by the sensations formerly so distressing to him.

* I have, for some years, been in the habit of prescribing this very active medicine in paralytic affections, and in certain diseases of debility, with very flattering promises of success.

him. I desired him to continue the medicine a few weeks longer.

On the 3d of June 1808, I was glad to be informed by my patient, that although the swelling of the spermatic chord still continued, yet his health was good, he was capable of exercising all his functions, and seldom had an emission.

### CASE VIII.

A gentleman aged 23, when about twelve years of age, fell prematurely into disgusting habits. About six months after the commencement of these practices, he felt very disagreeable sensations about his generative organs. This was not an acute pain, but a sensation as it were of something in the form of liquid constantly trickling about the perinæum and testicles. Soon after this, he experienced great pains shooting along the inside of his thighs, and from time to time, a sharp stinging pain darting along the penis. To these were added incontinence of urine, which, as it was passing, and for a few minutes after, caused a burning sensation with a sense of fullness about the glans penis. At this time he had frequent cold shiverings, with great restlessness during the day, and inability to sleep during the night, in consequence of being troubled by the most frightful dreams, the recollection of which, even while he was awake, terrified him. In this situation he continued two years, when, in addition to the above complaints, he had frequent involuntary seminal emissions. An extensive ulcer too broke out on the penis, which was healed up in a few weeks by simple dressings. His urine at that time became very turbid and extremely fœtid. The night-discharges continued to increase in frequency, and about a year after their commencement, he was affected with severe pains in the back and stomach, which spread to the intestinal canal, and he became very costive. Latterly these sensations had affected every part of his body, rendering even the friction of his wearing apparel painful to him. The great debility he now laboured under was indescribable, and the semen actually run from him on using the slightest motion. He was advised to use sea-bathing, a very common advice with medical men, when complaints are likely to baffle them; but from this he derived no benefit; even while making the exertion necessary in swimming, seminal emissions distressed him, and this was immediately followed by cold shiverings and grea[illegible] debility.

debility. The glans penis was swelled to an enormous size; and the preputium was pushed behind it. This continued several months, when the swelling began gradually to abate, but the prepuce never returned to its natural situation. This was soon succeeded by a continual perspiration all round the sacrum, and the parts immediately in its neighbourhood; and soon afterwards he experienced most excruciating pains in all the lumbar vertebrae, which were followed by an evident distortion of these bones. General emaciation, to a great degree, soon followed, and inflammation again affected the penis, but much more generally than on the former occasion. It, however, only continued a short time, when it disappeared spontaneously. An intolerably fœtid discharge of a yellowish colour now proceeded from the corona glandis, and he applied to a surgeon, who told him that his disease was venereal, and prescribed for him a course of mercury. From this he derived no advantage, although he continued to use it several weeks. He was then informed by his medical attendant, that his complaint was gravel, and advised to use sea bathing for its removal. His penis and testicles now shrunk very considerably, and were drawn up so close to the under part of his abdomen, as scarcely either to be seen or felt. Soon afterward, however, his testicles and scrotum swelled prodigiously, and ever since that time the convolutions of the epididymus have been enlarged and distinctly felt through the scrotum.

For several years past, most of the above complaints have been stationary. By degrees, however, his mind partook of the general disorder; he became very timorous, and the least alarm threw him into great agitation. He became excessively weak, and quite incapable of following any occupation: and night sweats, with difficulty of breathing, hoarsenes, and cough, prevented him from sleeping even when he became drowzy. Great depression of spirits, with languor, dimness of sight, tingling in the ears, and continual horror of mind, had for several years past been gradually added to his other complaints, which, when I first saw him (Nov. 1, 1807) had rendered his life a very great burthen to him.

I prescribed for him twenty drops of the tincture of cantharides, to be taken in a glass of water, four times a day. He gradually increased the dose, and at the end of four weeks, he was taking half an ounce of the tincture daily, when it, for the first time, occasioned him considerable pain in passing water. Being three or four miles distant, distant, I did not see him when this sensation was first produced, and, continuing to take the medicine in the same quantity, the pain occasioned was of course very severe. For two or three days, about that time, he passed water involuntarily, and an almost constant profuse perspiration pervaded his whole skin. I desired him to give up the use of the medicine for a few days, to apply warm cloths to the lower part of his belly, and to take a small purgative. In a few days these uneasy symptoms disappeared, and I desired him to recommence the tincture in small doses.

With the usual cautions, which I have elsewhere recommended, he continued to use the tincture, sometimes in larger, sometimes in smaller doses; and by the middle of January 1808, he became much stronger, took his food better, and some of his numerous complaints had entirely disappeared. Even at this cold season of the year, I desired him to use the cold bath, which, in addition to the former prescriptions, he continued to do with the greatest advantage. I desired him likewise to take half a pint of wine every day, and to live on good nourishing diet. Under the properly regulated management of these prescriptions, he recovered from his complaints by slow degrees, and he was, July 1808, stout and active; and but for that general gloom, which his former state of body seems to have entailed upon him, he has no complaint. I have desired him to decrease the doses of the cantharides by slow degrees, and to continue the cold sea-bathing for the remainder of the season.

In September 1808, this patient was not affected with any of the former symptoms of his complaint; the spermatic chord still remained enlarged, and he had occasionally emissions; but even these were so rare as not to constitute disease.

No. 4, *St. James's Street, Edinburgh.*

